# Cultural adaptation of a children’s weight management programme for Bangladeshi and Pakistani families in the UK: a cluster-randomised feasibility study protocol

**DOI:** 10.1186/s40814-016-0089-4

**Published:** 2016-08-12

**Authors:** Miranda Pallan, Tania Griffin, Emma Lancashire, Kiya Hurley, Jacqueline Blissett, Emma Frew, Paramjit Gill, Laura Griffith, Karla Hemming, Kate Jolly, Eleanor McGee, Charlene Mulhern, Jayne Parry, Janice L. Thompson, Peymane Adab

**Affiliations:** 1 Institute of Applied Health Research, University of Birmingham, Edgbaston, Birmingham, B15 2TT UK; 2 School of Psychology, University of Birmingham, Edgbaston, Birmingham, B15 2TT UK; 3 School of Social Policy, University of Birmingham, Edgbaston, Birmingham, B15 2TT UK; 4 Birmingham Community Healthcare NHS Trust, St. Patrick’s Centre, Frank St, Birmingham, B12 0YA UK; 5 Birmingham Public Health, Birmingham City Council, 10 Woodcock St, Birmingham, B7 4BL UK; 6 School of Sport, Exercise and Rehabilitation Sciences, University of Birmingham, Edgbaston, Birmingham, B15 2TT UK

**Keywords:** Obesity, Children, Treatment, Weight management, Pakistani, Bangladeshi

## Abstract

**Background:**

Group-based children’s weight management programmes are widely available in the UK and evidence shows that these are effective in the short-term. No programmes have been specifically developed to meet the cultural requirements of UK minority ethnic communities. South Asian children are a high-risk group for obesity and its consequences; therefore, the study aim is to adapt an existing weight management programme for children aged 4-11 years and their families to ensure cultural relevance to Pakistani and Bangladeshi communities, and undertake a feasibility study of the adapted programme.

**Methods/design:**

Pakistani and Bangladeshi families of overweight children who have been offered the existing children’s weight management programme in Birmingham, UK, will be invited to interviews and focus groups to explore their experiences and views of the programme. These data, together with existing literature and service provider information, will inform adaptation of the programme to be more culturally relevant to these families. The feasibility study will employ a cluster-randomised design, and will assess success of programme adaptation and feasibility of programme delivery. Planned programmes will be randomised to be delivered as the adapted programme (intervention) or the standard programme (comparator) with a 2:1 ratio. The primary outcome will be the proportion of Pakistani and Bangladeshi families completing the adapted programme. To assess recruitment, retention and data collection methods to inform a future trial, we aim to recruit 80 participants. A range of assessments will be undertaken with participants pre-, post- and 6-months post-intervention.

**Discussion:**

This study addresses the identified need to provide children’s weight management programmes that are suitable for minority ethnic communities. Whilst the focus of the intervention adaptation is on Pakistani and Bangladeshi communities, the programme will be developed to be flexibly delivered to meet the cultural needs of communities of all ethnic compositions. The feasibility study will directly compare the adapted and existing weight management programmes, and will enable a comprehensive evaluation of the success of the adaptation. Essential information will also be gathered to inform the design and sample size calculation of a future trial to evaluate intervention effectiveness.

**Trial registration:**

ISRCTN81798055, registered: 13/05/2014.

## Background

In England, 19 % of 10–11 year olds are obese and a further 14 % are overweight [1]. South Asian children (persons originating from the Indian subcontinent, including Pakistan and Bangladesh) have a higher prevalence of obesity compared with white children (24 vs. 18 % in 10–11 year olds). Specifically, at age 10–11 years, Pakistani and Bangladeshi children have higher obesity levels than their white British counterparts (28 and 33 % in Pakistani and Bangladeshi boys compared with 19 % in white British boys, and 22 % in Pakistani and Bangladeshi girls compared with 16 % in white British girls) [1]. Obesity in childhood is associated with a range of short and long-term physical, psychological and social consequences [2], as well as higher risk of obesity in adulthood [3]. South Asians comprise the largest minority ethnic population in the UK and are a particularly vulnerable group with regard to obesity and its health consequences. In addition to having higher adiposity than white European populations [4], South Asian adults are more likely to suffer the cardiometabolic consequences of obesity compared with other ethnic groups in the UK, and even in childhood, South Asians have been shown to have increased markers of cardiovascular risk [5, 6]. Thus, South Asians are an important target group for obesity intervention at all ages.

To date, evidence for effective childhood obesity treatment programmes is limited. In the Cochrane review conducted by Oude Luttikhuis et al., a meta-analysis of a subset of studies showed that lifestyle intervention programmes do have a small but clinically meaningful effect on weight status at 6 months follow-up (a reduction in BMI z-score of 0.06 and 0.14 compared to standard care in the preadolescent and adolescent age groups, respectively) [7]. However, the review also highlighted methodological issues in the included studies such as insufficient power, lack of allocation concealment, high attrition rates and lack of intention to treat analysis, so firm conclusions cannot be drawn from this review. Despite this, more recent randomised controlled trials (RCTs) of group-based, behavioural childhood obesity treatment programmes have also reported clinically meaningful effects on weight status and waist circumference [8, 9], although there is little evidence to support sustained effects for more than 12 months [10]. The absence of data on cost-effectiveness of childhood obesity treatment interventions has also been highlighted [7, 11].

Although the most effective intervention components are as yet undetermined, the available evidence suggests that in the preadolescent age group, interventions that address both diet and physical activity include behavioural elements and involving parents are most promising [7, 12]. A review on behalf of the American Heart Association also suggests that greater parental involvement in programmes leads to better long-term weight outcomes, although the specific nature of this parental engagement still needs to be determined [13].

A further limitation of existing evidence is the paucity of studies evaluating the effectiveness of childhood obesity treatment programmes in minority ethnic populations, particularly in settings outside the US [14–17]. Studies that have been undertaken have evaluated existing programmes with adaptations such as delivery of materials in different languages, tailoring of nutritional content and ethnic matching of programme providers. Two RCTs which evaluated culturally adapted interventions, one targeting Chinese American children aged 8–10 years [15] and the other a mixed population of Hispanic, black and white children aged 8–16 years [16], have reported small to moderate reductions in BMI z-score in the intervention compared with the control groups and effects appeared to be sustained for at least 8 months. In the UK, one small RCT involving 72 obese children has been undertaken to evaluate the effectiveness of a family-based behavioural treatment programme in an ethnically and socioeconomically diverse community of 8–12 year old children (43 % non-white European). The programme, originally developed in the US [18], was not culturally adapted, and whilst acceptable to the target population [19], it was not found to have a significant effect on weight [20].

To date, theoretical approaches to the cultural adaptation of childhood obesity treatment programmes have been lacking [14], as have studies to specifically evaluate the success of cultural adaptation. In their evidence synthesis review on the adaptation of health promotion programmes for minority ethnic groups, Liu and colleagues emphasised the requirement for future trials to compare culturally adapted programmes to standard programmes [21].

### Provision of childhood obesity treatment services in the UK

Although there is widespread provision of childhood obesity treatment services across the UK [22], there are no programmes that have been specifically designed to meet the needs of ethnically and culturally diverse populations, such as those found in many of the UK’s larger cities. Birmingham, the UK’s second largest city, is super-diverse with 42 % of the total population and 59 % of the 0–15 year old population from minority ethnic groups. Over half of these are from the Indian subcontinent [23]. A child weight management programme, First Steps, is available in Birmingham with over a third of referrals from Pakistani and Bangladeshi communities. However, inter-ethnic differences in completion rates (calculated from service attendance data) suggest that it is less well suited to families from these communities. Of those who start the programme, 40 % of Pakistani and Bangladeshi families complete it compared with 65 % of white British and African-Caribbean families.

Although there might be multiple reasons for this differential completion, it may in part be due to aspects of the current programme not being culturally relevant to Pakistani and Bangladeshi families. The existing weight management programme has developed and evolved over time based on available evidence as well as service provider experience to try to make it more acceptable to the local community. Prior to the introduction of First Steps in Birmingham, established and evidence-based child weight management programmes developed in other settings, such as MEND [8] and Watch It [24], had been delivered, but were found not to be suited to the local population. Specifically, the intensity and duration of the courses resulted in very low uptake rates and high attrition, and the lack of flexibility to change the structure and content of the programmes to accommodate cultural and language requirements was an issue. The structure, timing, duration and content of First Steps has been designed to be more acceptable to the local population, including a greater focus on parental engagement, greater interactive and visual programme content and the provision of interpreters. Elements of the Watch It and MEND programmes that worked in the local population were retained in First Steps (specific behaviour change strategies, and some content relating to nutrition and physical activity), and it was ensured that other elements that were incorporated were coherent with the evidence base. First Steps is achieving an average reduction in BMI z-score of 0.1 at programme end among children of families who complete the programme, which is comparable with effect sizes reported in clinical trials among programme completers. It was therefore deemed appropriate to build on this programme. The focus of this study is to further develop and culturally adapt the current First Steps programme, using a theoretically informed approach, such that it better meets the needs of these communities. However, a programme specifically for Pakistani and Bangladeshi families would not meet the wider needs of a super-diverse community, such as Birmingham. Super-diversity is characterised by overlapping factors including country of origin, ethnicity, language, religion, regional/local identities, migration history and experience, and immigration status [25, 26]. This complexity within the population creates a challenge regarding how we respond to meet the health needs of all members of society. Thus, the study aim is to develop a culturally adapted programme which is flexible enough to accommodate the needs of all families that attend, and can be transferred to communities with a different cultural and ethnic composition. To ensure the developed programme is suitable for all, we will evaluate it in a feasibility study involving families from various ethnic and cultural backgrounds.

### Study aims and objectives

The study is planned in two phases. In the first phase, we aim to adapt a weight management programme for children aged 4–11 years and their families, so that it is tailored to be culturally relevant to Pakistani and Bangladeshi communities, but appropriate for families from all ethnic and cultural communities. In the second phase, we aim to undertake a feasibility study of the culturally adapted intervention programme.

Specific study objectives are:

1. To explore factors which promote or discourage engagement with and completion of existing childhood obesity treatment programmes among Pakistani and Bangladeshi families.
2. To use this information together with existing research evidence to develop a culturally tailored, theoretically informed childhood obesity treatment programme.
3. To assess the feasibility of delivery of the new programme.
4. To assess the proportion of Pakistani and Bangladeshi families, and proportion of all families completing the culturally adapted intervention programme.
5. To assess acceptability of the programme to Pakistani and Bangladeshi families, and families from other ethnic groups.
6. To assess the feasibility of participant recruitment, randomisation and follow-up and collection of outcome data to inform a future trial evaluating intervention clinical and cost-effectiveness.
7. To collect data on recruitment, attrition and relevant outcome measures to inform parameters of such a future trial.

## Methods/design

### Study design

**Fig. 1:**
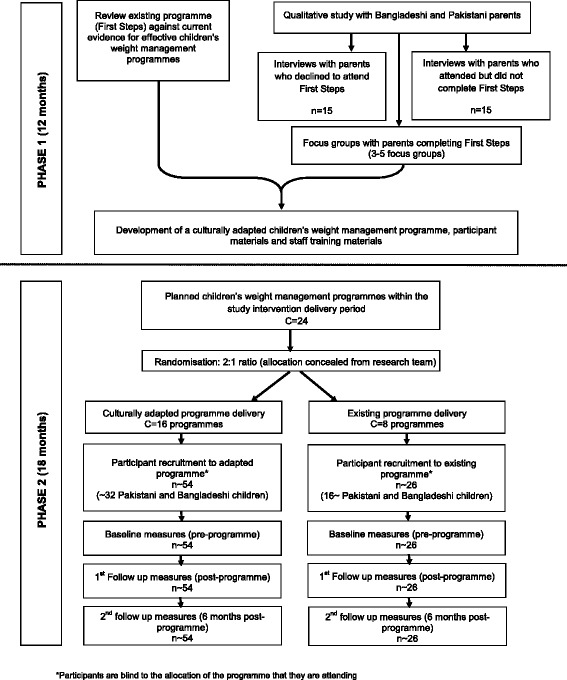
CHANGE study flow chart – study design and participants

The first phase addresses the theoretical and modelling stages of the UK Medical Research Council (MRC) framework for the development and evaluation of complex health interventions [27, 28] and involves adaptation of the current children’s weight management programme delivered in Birmingham. The adaptation process will be informed by evidence for effective childhood weight management intervention, and the experiences and perceptions of Pakistani and Bangladeshi families who have participated in or declined to participate in the current programme. The second phase is a two arm cluster-randomised feasibility study that compares the adapted programme to the existing programme, addressing the feasibility stage of the MRC complex health intervention framework. Data will be collected from participants at three time points: before programme attendance, after programme completion and 6 months after programme completion. A summary of the study design is shown in Fig. 1.

### Setting

The proposed study takes place in the UK’s second largest city (Birmingham) with a population of nearly 1.1 million; 16.5 % from Pakistani and Bangladeshi communities. Children from these communities comprise 26 % of the Birmingham population aged 0–15 years [23].

First Steps, the children’s weight management programme to be adapted, is commissioned by Birmingham City Council and delivered by Birmingham Community Healthcare NHS Trust. All families in Birmingham with a child of primary school age (4–11 years) who has a BMI over the 91st centile of the UK 1990 growth reference charts [29] are eligible to attend First Steps. Referral pathways to the programme include through health professionals, schools, the National Child Measurement Programme (NCMP; a BMI measurement programme for all school children aged 4–5 years and 10–11 years), and self-referral. First Steps is a group-based programme delivered as seven one-hour sessions over 6–7 weeks in community venues. Session content covers nutrition education, behaviour change and physical activity promotion. Parents/carers attend all sessions and children accompany them for the first and last sessions, when children’s heights and weights are measured. Participants for both phases of the study will be identified through the current children’s weight management services. The existing service infrastructure will be used to deliver the intervention and comparator programmes in the phase 2 feasibility study.

### Phase 1: adaptation of the First Steps children’s weight management programme

#### Information sources

Several sources of information will be used to inform adaptation of the children’s weight management programme: (1) qualitative data from Pakistani and Bangladeshi parents/carers of overweight children; (2) current evidence relating to children’s weight management; and (3) local information from the current children’s weight management service.

### Interviews and focus groups with Pakistani and Bangladeshi parents of overweight/obese children

Pakistani and Bangladeshi parents who have agreed to attend the First Steps children’s weight management programme in the previous year will be invited to participate in this part of the study. Three groups will be identified: (1) those who were offered the First Steps programme but declined to attend; (2) those who attended but did not complete the programme (attended less than 60 % of the programme); and (3) those who completed the programme. Parents/carers in the third group who completed First Steps will be invited to participate in focus groups. The focus group method explicitly uses group interaction as a way of stimulating discussion, resulting in the generation of richer data [30]. Whilst focus groups are the preferred method it is recognised that recruitment of participants in the first two groups is challenging, as they have not engaged with the First Steps programme. Therefore, we will undertake individual in-depth interviews (face-to-face preferred, but telephone interview offered) to maximise participation. We aim to recruit 15 participants in each of the first two groups to take part in interviews, and hold three to five focus groups in community venues with the third group of participants. Parents/carers in the third group who wish to participate but cannot attend a focus group will be offered an interview.

Potential participants will be approached initially by the children’s weight management service through a telephone call. If they express an interest in participating, a participant information leaflet will be posted to them and they will receive a follow-up telephone call to arrange an interview or focus group. Signed consent will be obtained at the time of the interview or focus group, and participants will receive a £10 shopping voucher.

The interviews and focus groups will be undertaken either by core members of the research team with experience of undertaking qualitative research (if the participant’s preferred language is English) or by Community Researchers. These are members of the UK Pakistani and Bangladeshi communities who have undergone bespoke training to enable them to undertake qualitative data collection in this study, and who can speak relevant languages and understand the cultural context of participating families. Both core and Community Researchers will record their observations and reflections after each interview or focus group, to provide context to local and cultural understandings and references.

Semi-structured interview and focus group schedules will be developed, informed by literature and input from the study Parent Advisory Panel (comprising Pakistani and Bangladeshi parents). In addition to a general exploration of participants’ experiences of the First Steps programme, the specific research questions to be explored are shown in Table 1.

**Table 1:** Research questions explored in phase 1 interviews with Pakistani and Bangladeshi parents of overweight and obese children

•	What are the barriers and facilitators to participating in and completing the programme?
•	Which aspects of the structure, content and delivery of the programme are perceived as problems?
•	What aspects of the structure, content and delivery of the programme are valued?
•	What information, content or format would increase the appeal of the programme?
•	What might need to change about the current programme to ensure its cultural relevance?

Interviews and focus groups will be audio-recorded and transcribed for analysis verbatim. Those undertaken in languages other than English will be translated and transcribed by the Community Researchers. Data analysis will be systematic and iterative, based on the constant comparative method [31]. Open coding of a selection of transcripts will be undertaken to identify initial key themes and emerging patterns, enabling development of a coding frame which will be refined and applied to the full dataset.

### Review of children’s weight management intervention literature and guidelines

The UK National Institute for Health and Care Excellence (NICE) published a comprehensive evidence review and guidelines on managing overweight in children and young people in 2013 [32]. In addition, more recent evidence on effective children’s obesity interventions [10, 33–36] and on behaviour change techniques that are effective in influencing obesity-related behaviours in children [37] have been identified. The research evidence and guidelines will be considered throughout the adaptation process to ensure that the developed programme incorporates approaches that have been acknowledged to be effective and that the structure, content and delivery is consistent with current guidance.

### Information from the existing children’s weight management service

Direct observation of the current children’s weight management programme will be undertaken to assess how the structure, content and delivery work in practice. In addition, all staff involved in service planning, recruitment and delivery will be consulted so that a clear picture of the existing infrastructure and processes is gained, and current issues with the programme are highlighted. Further discussion with relevant service provider staff will be needed during the adaptation process, especially in relation to any proposed changes that may have an impact on the current service infrastructure and provision.

### Process of intervention development

To guide the intervention adaptation process, The Behaviour Change Wheel (BCW) framework will be used [38]. This has been developed from 19 behaviour change frameworks, thus incorporating a broad range of drivers of behaviour (e.g. individual perceptions and beliefs, unconscious biases and the social environment). The target behaviours that require change are identified as: (1) programme attendance and engagement; (2) dietary intake; and (3) physical activity. The capability, opportunity, motivation and behaviour (COM-B) model at the centre of the BCW will be used to specifically identify what needs to change. For each target behaviour the data gained from interviews and focus groups will be mapped to the three aspects of the COM-B model: capability (physical or psychological), opportunity (physical or social) and motivation (reflective or automatic). Once it is understood what needs to change, intervention functions that are likely to address the specific aspects of capability, opportunity and motivation will be identified from the BCW framework and incorporated into the intervention programme.

### Cultural adaptation

To address specific cultural adaptation, another theoretical development process will be employed in parallel. In a 2012 report on the cultural adaptation of health promotion programmes for minority ethnic groups, Liu and colleagues proposed a 46-item typology of cultural adaptation based on a comprehensive systematic review of health promotion programmes adapted for minority ethnic groups. They proposed that this typology could be used together with a generic programme theory to guide development of culturally adapted interventions [21]. The programme theory describes the processes within the health promotion intervention cycle: conception/planning, promotion, recruitment, implementation, retention, evaluation, outcome and dissemination. Application of this programme theory ensures that all aspects of the programme are considered during the adaptation process. Therefore, the interview and focus group data will additionally be used to identify appropriate types of adaptation listed in Liu’s Typology and the relevant programme theory stage that each adaptation type maybe applied to will also be identified from this data. In this way, we will identify specific opportunities for cultural adaptation.

### Detailed planning of the adapted programme

Identification of the relevant intervention functions to address the behaviours that need to change, and the opportunity for cultural adaptation within the different stages of programme delivery will provide a guiding framework on which to build the detail of the intervention. During the detailed planning, we will ensure that the adapted programme allows flexibility in delivery such that it is appropriate for children of different ages, and is coherent with current evidence and guidelines. We will also use the information gained locally from observation and service provider consultation. The facilitators will be provided with an overall structure and format for the programme, the structure and content of each session, and a detailed delivery manual. Comprehensive training will also be provided.

### Phase 2: a feasibility study of the adapted children’s weight management programme

#### Design

The feasibility study will be conducted as a small scale cluster-randomised controlled trial. This will enable estimation of the proportion of families (both Pakistani/Bangladeshi families, and families of other ethnicities) completing the developed intervention programme. It will also provide key information for planning a future effectiveness and cost-effectiveness evaluation of the adapted intervention, including likely recruitment and attrition rates, optimal data collection methods, and parameters to inform a sample size calculation. Programmes delivered across Birmingham within the study period will be randomised to be delivered as either the adapted programme (intervention) or the standard programme (comparator). Families referred to the children’s weight management service will not be aware of the allocation of the programmes and will attend the programme that is most convenient for them.

### Participants

Families in Birmingham with a child age 4–11 years who is overweight (defined as greater than 91st centile for BMI on the UK 1990 growth reference charts [29]) and who are referred to the existing children’s weight management service within the study recruitment period will be eligible to participate. Local service data indicate that 40 % of referrals are Pakistani and Bangladeshi families; however, as the intervention will be adapted primarily to suit Pakistani and Bangladeshi families, we aim to recruit 60 % of participants from these communities and therefore will prioritise recruitment of these families. This will enable us to explore the success of programme adaptation from the perspective of these families. Approximately 800 children and families of all ethnicities are referred to the First Steps programme annually. We aim to recruit 80 children and their families in a 6-month period, and therefore need to recruit 20 % of referred families to participate in the study.

Families referred to the weight management service will initially receive a study invitation letter and information leaflet from the service providers, which will be followed up by a telephone call. If they are interested in participating in the study, they will be contacted by a researcher to arrange a time for an initial home visit to gain consent and collect baseline data. Home visits are anticipated to maximise participation and assist in timely data collection. This data collection process will also allow for blinding of researchers undertaking data collection in a future trial. If a parent/carer requires communication in another language, a person who speaks the relevant language will contact them by telephone to explain the study and invite them to participate, and will complete the initial visit. Written consent will be gained from participating parents/carers, and assent will be gained from children (written if 8 years or more and verbal if under 8 years).

### Study arm allocation

Randomisation of the weight management programmes will be conducted by Birmingham Primary Care Clinical Research and Trials Unit at the beginning of the feasibility study to enable service providers to plan delivery of the programmes. Programme allocation will be concealed from the research team so that they unaware of this at the time of baseline data collection. During the feasibility study period, delivery of 24 programmes across Birmingham is planned. The programmes will be randomised using a 2:1 ratio, so 16 programmes will be the adapted programme and eight will be the standard programme. This will ensure a sufficient number of families in the intervention arm to enable calculation of the primary outcome of completion (see sample size calculation).

### Intervention

Families in the intervention arm will receive the weight management programme for children aged 4–11 years that is developed in phase 1 of the study.

### Comparator arm

Families in the comparator arm will receive the existing First Steps children’s weight management programme (usual care). To avoid contamination between the two arms, the standard and adapted programmes will be delivered by different facilitators.

### Evaluation of the success of intervention adaptation

The primary outcome for the evaluation of programme acceptability and success of its adaptation will be the proportion of Pakistani and Bangladeshi families completing the adapted programme. Proportion of families of all ethnicities completing the adapted programme will also be determined. Completion of the course is defined as attending at least 60 % of the course. This will be assessed using routine attendance data collected at each programme session.

Observations of programme sessions and interviews with the programme facilitators will aim to assess the success of the adaptations in addressing the previously identified barriers and ease of implementation. Each facilitator will be observed delivering all sessions at least once across the study intervention period. Members of the research team will undertake observations and will make notes on the delivery of and response to the session. Additionally, in depth interviews will be undertaken with parents/carers who attend the adapted programme to explore their experiences of the programme, barriers and facilitators to engagement and their views on its structure, delivery and content. Approximately 15–20 interviews will be undertaken (approximately half with Pakistani and Bangladeshi parents and half with parents of other ethnicities). We will purposively sample to include parents who completed the programme and those who only partially attended. Interview schedules will be developed to include prompts on the specific adaptations made during the programme development process. The interviews will be undertaken by either the core research team or Community Researchers in the participants’ preferred languages. The interviews will be audio-recorded, translated and transcribed verbatim. Views of children completing the adapted programme will also be sought. Approximately ten children aged 8 years or over (five Pakistani/Bangladeshi and five of other ethnicities) will be recruited to participate in interviews to explore their experiences and engagement with the programme, using appropriate interview techniques for this age group. Written consent and assent will be gained from facilitators, parents and children for participation in the interviews.

### Outcome measures for use in a future trial to evaluate programme clinical and cost-effectiveness

A range of other outcome measures will be assessed for feasibility and thus inform design of a definitive effectiveness and cost-effectiveness evaluation. Child measures will include measures of adiposity, assessment of pubertal status, usual eating patterns, objectively measured physical activity, psychosocial measures and a quality of life utility measure. Measures with parents and other family members will include adiposity measures, assessment of family eating and physical activity behaviours, parenting styles, parental feeding practices and parental self-efficacy. Further details of these assessments are shown in Table 2.

**Table 2:** Outcome measures on children, parents and other family measures in the phase 2 feasibility study

Measures of adiposity	
•	Height and weight - to calculate BMI z-score
•	Waist circumference
•	Percentage body fat - derived from bioimpedance analysis
Pubertal assessment	
•	In girls aged > 8 years - visual assessment of breast development (child clothed): a modified approach to the Tanner breast development scoring system [44], and history of menarche obtained
•	In boys - assessment of facial hair growth
Usual eating patterns	
•	Children’s Dietary Questionnaire administered to parents/carers [45] - adapted for use in the local population
Objectively measured physical activity	
•	Geneactiv (wrist) or Actigraph (hip) worn for 7 days
Psychosocial dimensions	
•	Health-related quality of life - assessed using Pediatric Quality of Life Inventory [46]
•	Body image - assessed using the Figure Rating Scale [47] (adapted for use in the local population)
Quality of life utility measure for use in economic evaluation	
•	Child Health Utility 9D (CHU 9D) [48]
Parent/carer and family assessments	
•	Height, weight and bioimpedance of parents/carers and other family members - to calculate BMI and percentage body fat
•	Parent/carer feeding practices - assessed using the Comprehensive Feeding Practices Questionnaire [49]
•	Parenting style - assessed using the Parenting Styles and Dimensions Questionnaire [50]
•	Parental self-efficacy - assessed using the Parental Locus of Control Scale [51]
•	Family food and physical activity behaviours - assessed using the Family Nutrition and Physical Activity screening tool [52]

### Sociodemographic information

Age, sex, and ethnicity of the child, family migration history, religious beliefs/practices, home postcode (to be mapped to Index of Multiple Deprivation scores), parent/carer occupation, and parent/carer education level will be collected from parents/carers at the baseline home visit.

### Outcome data collection procedure

Physical measurements and other assessments will be undertaken on children and parents/carers at three time points: baseline, post-intervention and 6 months post-intervention. Trained research staff will undertake data collection in the participant’s home, or at another location convenient to the participant’s family. For the questionnaire-based data collection researchers will verbally administer questionnaires directly to children. Parents can choose to self-complete or verbally complete questionnaires with the researcher. If the participant’s preferred language is not English, trained Community Researchers will administer the questionnaires and undertake other data collection using the relevant language. Researchers and families will be blind to their treatment allocation at the time of baseline measures. The feasibility of this data collection approach will be assessed to inform a future trial.

### Cost data to inform a future economic evaluation

Methods for collecting data on direct intervention costs and costs from a societal perspective will be developed and tested during the feasibility study. Specifically we will include items such as parent/carer productivity costs (time off work to attend the intervention sessions), associated childcare costs and changes to the weekly food bill.

### Process evaluation

We will develop process evaluation methods to inform a future trial, in accordance with the UK MRC guidance on process evaluation of complex interventions [39]. The planned interviews with parents/carers attending the programme, direct observation of programme delivery, and interviews with programme facilitators will enable assessment of various aspects of implementation, mechanisms of impact and contextual factors influencing intervention delivery and how the intervention is received. Specifically, direct observation and interviews with facilitators will enable assessment of dose and fidelity of delivery, and direct observation and interviews with parents and children will enable assessment of participant response, unanticipated pathways and contextual influences.

### Sample size

To calculate the primary outcome (the proportion of Pakistani and Bangladeshi families completing the adapted programme), we will use anonymised data on completion that is routinely collected by the children’s weight management service. From existing service data, the mean group size at the start of a programme is 11. Therefore, we expect approximately 176 families to start attending the 16 adapted programmes delivered in the intervention period, 40 % of which will be Pakistani or Bangladeshi (i.e. approximately four to five Pakistani and Bangladeshi families will start attending each of the adapted programmes within the study period, 70 families in total). This will allow an estimation of the proportion of Pakistani and Bangladeshi families completing the programme to within 26 % precision. For example, if 65 % of Pakistani and Bangladeshi families complete the adapted programme, the 95 % confidence interval of the estimate of completion will be 52–78 %. To estimate this confidence interval, we have inflated the variance we would expect under individual randomisation by the typical variance inflation factor for cluster trials (with an additional inflation of 11 % to account for the varying cluster size [40]). We have assumed a mean cluster size of 5. There is no specific data to inform the estimate of the intra cluster correlation co-efficient (ICC), therefore, using current recommendations we have drawn on patterns of ICCs from other sources [41]. Our outcome is a process outcome; our clusters are small; and the prevalence of our outcome (completion) is expected to be moderate. The latter is associated with generally lower ICCs whereas the two former are generally associated with higher ICCs. We therefore have assumed a moderate ICC of 0.05 [42].

To test recruitment and data collection methods and gain an estimate of recruitment and attrition rates to inform a future trial, we plan to recruit at least 80 children and families into the study. This number of participants will also enable estimation of the key parameters required for a sample size calculation of a future trial [43].

### Data analysis

The proportion of Pakistani and Bangladeshi families completing the adapted intervention programme and 95 % confidence intervals will be calculated using routinely collected service data. Secondary analyses will include estimation of the proportion of all families completing the adapted programme and 95 % confidence intervals, and a comparison of proportion of families completing the programme in the intervention and comparator arms (using intention to treat analysis). Confidence intervals will be estimated from the appropriate mixed-effects model to allow for the clustered nature of the data.

To provide information on the feasibility of outcome measures, we will report the number of measurements attempted for each outcome and the proportion of complete assessments made. To provide sample size calculation parameters for a future trial to assess intervention effectiveness, feasible outcome measures will be reported using the appropriate summary statistics and correlations between baseline and follow-up will be estimated for continuous outcomes.

Although the study is not sufficiently powered to detect minimum clinical differences in adiposity outcomes between intervention and comparator arms, estimates of differences in these and other outcomes between arms will be calculated. Mixed-effects models will be developed, adjusting for clustering and baseline measures, and 95 % confidence intervals will be reported and differences deemed significant at the 5 % level. This model will also provide an estimate of the ICC and 95 % confidence interval for use in a sample size calculation for a future trial to evaluate effectiveness.

### Research governance

The study is funded by the UK National Institute for Health Research Health Technology Assessment programme, and is sponsored by the University of Birmingham. Ethical approval was obtained in July 2014 from Edgbaston NHS Research Ethics Committee, West Midlands, UK (14/WM/1036). The study is overseen by an externally appointed Study Steering Committee comprising three subject experts (two Public Health specialists with an interest in childhood obesity prevention and management, and an expert on equality and diversity in relation to health and care) and a public representative.

All data are stored securely and confidentially in accordance with University of Birmingham data protection policies. Feasibility study data are entered into a secure database, developed and maintained by the Birmingham Primary Care Clinical Research and Trials Unit.

## Discussion

This study attempts to address the important issue of the provision of children’s weight management services that are relevant to all ethnic and cultural communities within the UK. There is little existing research on culturally adapted children’s weight management interventions [14–16], and none based in the UK. Whilst the focus of the phase 1 intervention adaptation is to ensure the children’s weight management programme is more suitable and engaging for Pakistani and Bangladeshi families, the overall aim is to ensure the developed programme is adaptable to the needs of families from all communities. If programme adaptation successfully increases the likelihood of programme completion by families, then this programme has the potential to be rolled out across the UK after evaluation to assess programme effectiveness.

The intervention adaptation process uses two theoretical frameworks, each fulfilling a specific purpose. The BCW [28] guides the theoretical development of a complex intervention, enhancing the capabilities, opportunities and motivation to change target behaviours within families attending the programme. The typology of cultural adaptation and health promotion programme theory [21] enables us to explicitly identify the adaptations required to ensure cultural barriers are minimised, and develop a programme that better meets the cultural needs of a wider audience.

A need for direct comparisons between existing health promotion programmes and those that are adapted for minority ethnic groups has been highlighted [21]. Phase 2 of this study directly compares the adapted programme to the standard programme, and evaluates the success of programme adaptation through several methodologies. The feasibility study also enables further refinement of the adapted programme and allows us to gather information to inform a trial evaluating programme effectiveness. This study will provide important information on both the feasibility of recruitment in a culturally and ethnically diverse population, and likely attrition rates in a trial. The process of home visits for data collection is a particular strength of the study design as it is likely to minimise attrition. A range of outcome measures that relate to the intervention are being tested in the feasibility study. This will enable identification of the primary outcome measure and important secondary outcome measures for inclusion in a future trial to evaluate programme effectiveness. We recognise that multiple outcome measures may overburden participants, and the feasibility study gives us the opportunity to assess this and prioritise which outcome measures to include in a future trial. The feasibility study also provides the opportunity to develop rigorous methodology for process and cost-effectiveness evaluation for use in a future trial. These are key areas to address in the definitive evaluation of complex interventions [27, 28].

The decision to undertake a full scale trial to evaluate clinical and cost-effectiveness of the adapted programme will primarily depend on the success of the programme at increasing family completion rates, and the acceptability of the programme to families from all cultural backgrounds. If we progress to a full trial, the valuable information gained on the feasibility of study design, processes and data collection will shape trial design and inform a sample size calculation.

### Study status

The study is registered on the International Standard Randomised Controlled Trial Register (ISRCTN81798055). Phase 1 was undertaken between September 2014 and August 2015. Participant recruitment for the phase 2 feasibility study commenced in September 2015 and is due to finish in April 2016, and final follow-up measures will be undertaken by October 2016.

## Abbreviations

BCW, Behaviour Change Wheel; BMI, body mass index; COM-B, capability, opportunity, motivation and behaviour; MRC, Medical Research Council; NCMP, National Child Measurement Programme; NHS, National Health Service; RCT, randomised controlled trial
